# A Stable C7 Fracture in a High School Quarterback: A Case of Clay Shoveler's Fracture

**DOI:** 10.7759/cureus.111440

**Published:** 2026-06-24

**Authors:** Kasey Stickler, David Rupp, Lauren Baumgartner, Charles A Gilliland, Katherine D Redmond

**Affiliations:** 1 Family and Community Health/Sports Medicine, Joan C. Edwards School of Medicine at Marshall University, Huntington, USA; 2 Family and Community Medicine/Sports Medicine, Joan C. Edwards School of Medicine at Marshall University, Huntington, USA; 3 Family Medicine, Joan C. Edwards School of Medicine at Marshall University, Huntington, USA; 4 Orthopedics/Sports Medicine, Joan C. Edwards School of Medicine at Marshall University, Huntington, USA; 5 Sports Medicine, Joan C. Edwards School of Medicine at Marshall University, Huntington, USA

**Keywords:** cervical spine injury, clay shoveler’s fracture, contact sports, spinous process fracture, sports-related injuries

## Abstract

Fractures of the lower cervical spinous processes, also known as Clay Shoveler’s fractures, are uncommon injuries typically associated with high-impact trauma but may also occur in contact sports. These avulsion-type fractures result from one of several mechanisms, including forceful muscle contraction, hyperflexion-hyperextension, or direct trauma at the cervicothoracic junction. This study presents the case of an 18-year-old football player who presented to a sports medicine clinic four days after a game-related neck injury with gradually improving pain managed conservatively with over-the-counter medications and ice. Physical examination revealed focal tenderness over the cervical seven (C7) spinous process with preserved strength, sensation, and range of motion. Radiographs confirmed an isolated C7 spinous process fracture. The patient was managed nonoperatively with immobilization in a rigid cervical collar, rest, and nonsteroidal anti-inflammatory drugs, followed by gradual weaning of the collar in coordination with neurosurgery. This case highlights the importance of considering Clay Shoveler’s fractures in athletes with localized cervical tenderness after contact injury and reinforces the efficacy of conservative management and multidisciplinary follow-up in achieving full recovery.

## Introduction

A Clay Shoveler’s fracture is characterized as an avulsion fracture of the spinous process in the lower cervical or upper thoracic vertebrae, with the term 'Clay Shoveler’s fracture' originating from its common occurrence among 20th-century construction workers employed in shoveling clay [1,2]. An avulsion fracture occurs when a bone is pulled from its normal position by a forceful contraction of attached muscle or tendon or by sudden tensile forces on a ligament. While this injury is not commonly reported, it can occur across all age groups, presenting in a bimodal distribution, frequently in individuals aged 15 to 24 years, and again in those aged 55 and older [3].

The frequently cited leading factors in identifying the primary contributors to injury are falls, automobile accidents, and sports [2,4]. In relation to this presented case, youth sports in the United States that pose the greatest risk for head and spine injuries include football, gymnastics, cheerleading, and ice hockey. Given that football has the highest participation rate among these sports, it also reports the highest number of severe injuries. Furthermore, in older individuals or those who suffer from osteoporosis, even mild trauma can lead to degeneration, causing the avulsion of the posterior structures during extension movements [5]. Multiple studies found the prevalence of Clay Shoveler’s fractures to be as low as 3.7% among trauma patients, with 41.9% requiring operative management [6].

Following the occurrence of this injury, patients typically present with central lower cervical pain, which may radiate toward the upper back. The pain often develops gradually and is described as both sharp and dull in nature [1]. Tenderness over the cervical seven (C7) spinous process may also be observed. The distinctive characteristic of this injury lies in the absence of instability, setting it apart from other types of spinal injuries that typically present with more severe neurological or musculoskeletal consequences. Given the typical presentation and conservative management of Clay Shoveler's fractures, the following case highlights a unique instance of this injury, offering further insight into its clinical course and treatment outcomes.

## Case presentation

The patient is an 18-year-old male football player who presented with neck pain following an injury sustained during a football game four days prior. During the game, he was tackled to the ground by another player, causing his helmet to be pulled off. As a result, his neck was hyperextended and twisted. The injury occurred near the end of the first half, and although he briefly exited the field due to the loss of his helmet, he was able to return and finish the half. At halftime, he took ibuprofen for pain and continued to play for the remainder of the game.

Initially, his pain was severe on the day of injury and following day but began to improve with over-the-counter Aleve/Tylenol and the use of ice over the following week. The patient reported a history of cervicalgia following a cervical spine sprain during a previous injury while wrestling in December 2019. At that time, an X-ray was performed, which was negative, and he was referred to physical therapy. However, his course of therapy was abbreviated due to the onset of the COVID-19 pandemic. A year later, in December 2020, he was seen again for similar symptoms and returned to PT for cervicalgia.

In May 2021, an MRI of the cervical spine was performed due to persistent symptoms. The results, reviewed by both radiology and sports medicine, revealed a mild amount of cervical stenosis, which did not meet the criteria for pathologic stenosis based on the Torg ratio. There was no appreciable compression or nerve irritation, and no acute fractures were noted. The patient was evaluated once more in January 2022 and was treated with a Medrol dose pack. He also completed a third course of physical therapy.

Since then, the patient reported that his neck had been aggravated a few times, but nothing as severe as the current episode. The acute pain, which began after the recent football injury, was localized over the spinous process of C7 and radiated to the left side. He denied any radicular symptoms.

Upon inspection, there was no obvious deformity, swelling, or bruising noted in the cervical spine. On palpation, tenderness was noted along the paraspinal musculature bilaterally, with more pronounced tenderness on the left side at the level of C6-C7. Additionally, tenderness was noted over the C7 spinous process. The patient demonstrated a full passive range of motion in flexion, extension, side bending, and rotation, without signs of restriction. Strength testing revealed 5/5 strength in both upper extremities bilaterally. Spurling's test was deferred due to localized tenderness over the C7 spinous process. Neurological examination showed that sensation was intact to light touch, pulses were two+ bilaterally, and upper extremity reflexes were two+ on both sides. Special tests, including the OK sign, thumb abduction, thumb distal interphalangeal flexion, metacarpophalangeal extension, and finger crossing, demonstrated range within normal limits.

**Figure 1:**
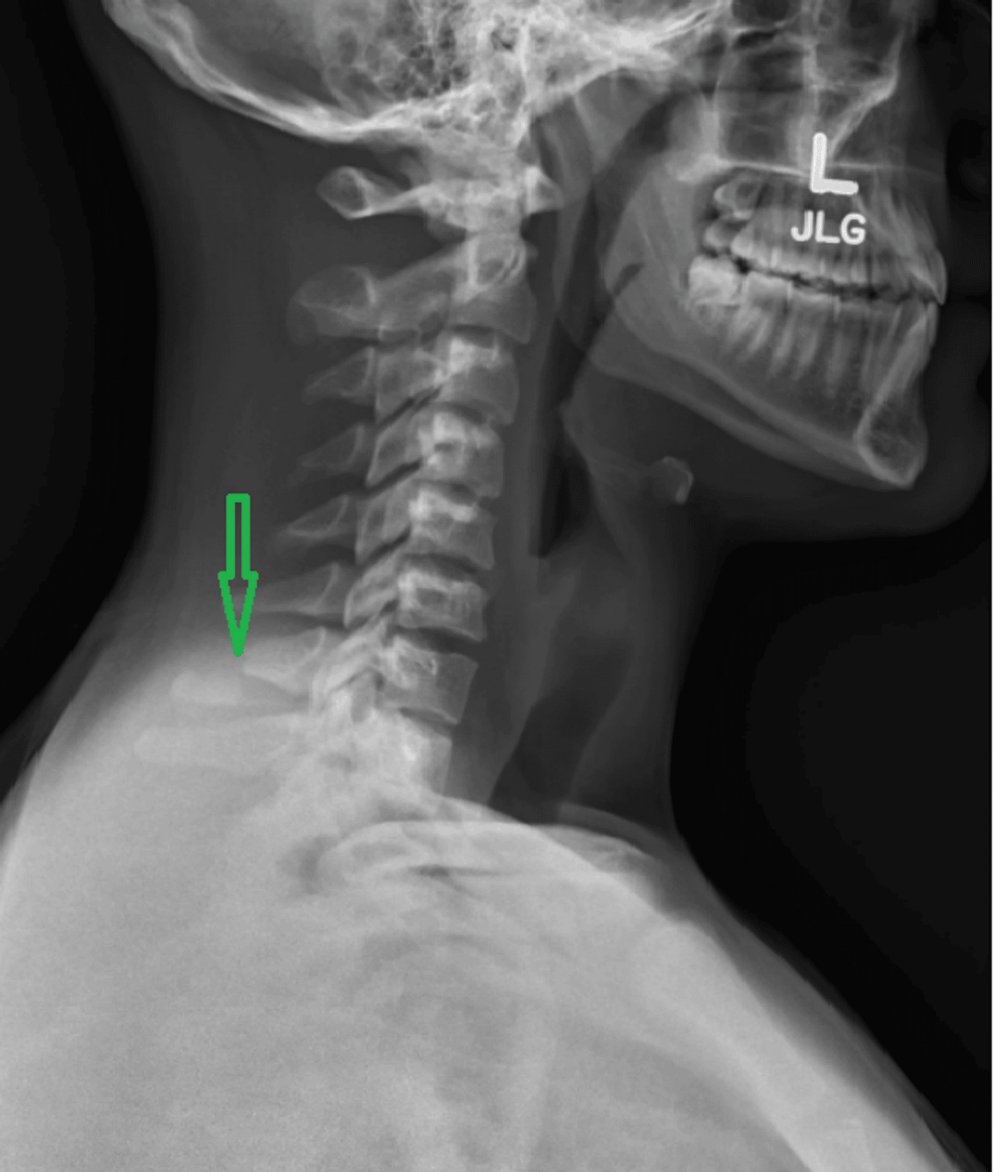
Clay shoveler's fracture The arrow indicates the C7 spinous fracture process.

After further testing, including a four-view cervical spine radiograph, a fracture of the C7 spinous process was identified (Figure 1). The neural foramina were patent, and there was a loss of normal cervical spine curvature, but no other abnormalities were noted. Based on all the above findings, the final diagnosis was confirmed to be a C7 spinous process fracture.

## Discussion

Given the overlap of symptoms that are commonly seen among other cervical spine conditions, a thorough differential diagnosis is essential to ensure the appropriate steps are taken. Possible conditions to consider include cervical strain, spinal cord neoplasms, vertebral artery dissection, spinal cord infections, and other cervical spine fractures [2].

Pathophysiology and diagnosis

The c-spine fracture can be attributed to four mechanisms: a fracture resulting from shoulder muscle contraction (involving the trapezius, rhomboid minor, and/or serratus posterior superior); a stress fracture caused by hyperflexion-hyperextension of the neck; an avulsion fracture associated with fracture-dislocations of the cervical spine; and a direct injury due to a deforming force applied to the cervical-thoracic junction [2]. Specifically, the head and upper cervical segments are subjected to forced flexion, counteracting the interspinous and supraspinous ligaments, due to shearing forces generated by the contraction of the upper back and neck muscles (e.g., trapezius and rhomboid). The resultant muscle forces act transverse to the axis of the spinous processes, thereby elevating the risk of overuse fractures [4,7].

In terms of diagnosis, the gold standard is a conventional spine radiograph. If poor visualization results from the lateral position, then swimmer’s view or a CT scan can be utilized [6]. MRI can then highlight the presence, if there is any, of spinal cord compression, edema, and/or hemorrhage [8]. In special circumstances, as with the osteoporotic patients, vertebral fracture assessment may be performed alongside bone mineral density scans [9].

Injury Classification 

Clay Shoveler’s fractures are classically described as avulsion fractures of the spinous processes of the lower cervical or upper thoracic vertebrae and most often occur as isolated injuries. Notably, there is no formal, injury-specific grading system for Clay Shoveler’s fractures described in the current medical literature [10]. Instead, these injuries are typically contextualized within broader cervical and thoracolumbar spine trauma classification frameworks, which are primarily designed to assess more complex or unstable spinal injuries [11].

Within the Subaxial Injury Classification (SLIC) system, which applies to injuries from C3 to C7, isolated spinous process fractures are generally categorized under compression-type injury morphology and would receive a low injury severity score, reflecting their stable nature [12]. Similarly, under the AO Spine classification system, such fractures would most closely align with minor type A (compression) injuries, although this system does not provide specific severity grading for isolated spinous process avulsion fractures [11]. As a result, clinical significance is not determined by formal grading criteria but rather by fracture location, the presence of single versus multiple spinous process involvement, degree of displacement, and associated symptom burden [12].

Differential Diagnosis

Although Clay Shoveler’s fractures are generally benign isolated avulsion fractures of the spinous process, differentiation from more complex posterior element fractures involving the lamina and spinal canal is vital. A critical imaging distinction is the presence of spinolaminar breach, defined as disruption of the cortical junction between the base of the spinous process and the lamina [13]. Identification of spinolaminar breach indicates a fracture extending beyond a simple avulsion pattern and suggests a more complex injury mechanism.

Spinous process fractures with spinolaminar breach have been associated with posterior ligamentous complex disruption, potential delayed instability, and an increased risk of neurological deficit [13]. These injuries may also be accompanied by nerve root avulsion, particularly in high-energy mechanisms, and therefore require different clinical consideration and management than an isolated Clay Shoveler’s fracture [13]. In the present case, imaging demonstrated an isolated spinous process fracture without evidence of laminar extension or spinolaminar disruption, supporting classification as a simple Clay Shoveler’s fracture and favoring conservative management.

This distinction is especially relevant given the anatomical overlap between the typical levels affected by Clay Shoveler’s fractures (C6-T3) and the origin of the brachial plexus (C5-T1). Preganglionic brachial plexus avulsion injuries represent a severe form of cervical spine trauma involving tearing of nerve roots from the spinal cord and may coexist with posterior element fractures [14]. Imaging findings associated with preganglionic nerve root avulsion include pseudomeningocele formation due to cerebrospinal fluid accumulation within the neural foramina, and in rare cases, epidural hematomas [14]. Delayed complications such as progressive myelopathy, developing months after injury, have also been reported and may result from spinal cord herniation, syringomyelia, superficial siderosis, or abnormal cerebrospinal fluid collections [14].

On computed tomography, a simple Clay Shoveler’s fracture appears as an isolated avulsion of the spinous process without extension into the lamina. In addition, preservation of the spinolaminar line on lateral radiographs and sagittal CT reformations can be appreciated [13]. In contrast, disruption of this line should prompt further evaluation for laminar involvement and associated soft tissue injury. CT with multiplanar reformations is the preferred modality for detecting spinolaminar breach and defining fracture extent [15]. In this patient, preservation of the spinolaminar line on CT supported the absence of complex posterior element injury and helped exclude higher-risk fracture patterns.

MRI is indicated when neurological symptoms are present, when CT demonstrates spinolaminar breach, or when clinical findings are disproportionate to radiographic appearance [13,15]. MRI is particularly valuable for identifying nerve root avulsions. This may be characterized by pseudomeningoceles and absent or disrupted nerve rootlets, as well as epidural hematoma, spinal cord edema or contusion, traumatic disc herniation, and posterior ligamentous complex injury [15]. For suspected preganglionic nerve root avulsion, MRI of the cervical spine is preferred over dedicated brachial plexus MRI, as it more reliably demonstrates pseudomeningocele formation and rootlet abnormalities [15]. The combination of pseudomeningocele and absent nerve rootlets has been shown to be highly specific for complete nerve root avulsion [16]. The absence of neurological deficits on serial examinations in this case reduced concern for occult nerve root injury and supported continued conservative treatment without advanced MRI at initial presentation.

Treatment and prognosis

In general, this injury occurs in isolation and is a non-operative condition, typically managed with conservative treatment, such as rest and physical therapy, and pain management with analgesics. A cervical collar may be worn as recommended by the provider or as needed [2]. Should a painful nonunion persist despite initial conservative treatment, surgical intervention involving the excision of the avulsed fragment may be warranted. Once the initial treatment plan is implemented, the prognosis largely depends on the response to conservative measures and the progression of healing. Most individuals fully recover and can return to their usual activities within three weeks to four months, typically without any ongoing restrictions. In rare cases, involving an unresolved nonunion, patients may endure ongoing dull pain in the cervical region long after the injury has occurred [1].

As it applies to the current case, the patient was fitted with a hard-sided cervical collar to immobilize the neck and provide support. He was instructed to refrain from all sports activities and was advised to avoid any actions requiring functional movement, including driving, to prevent further strain or injury. At the family's request, the patient was referred to a spine specialist for further evaluation. A follow-up appointment was scheduled for one week later. At the one-week follow-up with sports medicine, the patient was neurologically intact and displayed no significant cervical spine tenderness. The recommendation was made to limit the use of the cervical collar to daytime hours only. During a follow-up with neurosurgery later that week, repeat X-rays were performed, and the patient was cleared to transition out of the collar, using it as needed. A plan was established for a follow-up appointment in four weeks, with repeat X-rays scheduled.

## Conclusions

Clay Shoveler’s fractures, though uncommon, should remain an important diagnostic consideration in athletes presenting with focal cervical tenderness following contact or hyperextension injuries. Their stable nature and characteristic presentation allow for timely identification using standard radiographic evaluation, which is essential to distinguish them from more serious cervical pathologies.

This case highlights the characteristic recovery trajectory of a Clay Shoveler’s fracture, a typically non-operative injury with a favorable prognosis. The unique aspect of this case lies in the patient's relatively quick recovery and the careful management of his symptoms, which allowed for early collar reduction and a positive outlook for full functional return with no evidence of sustained neurological dysfunction or physical sequelae.
